# One-year regional brain volume changes as potential predictors of cognitive function in multiple sclerosis: a pilot study

**DOI:** 10.1007/s11845-023-03528-x

**Published:** 2023-09-29

**Authors:** Torcato Meira, Ana Coelho, Seyda Onat, Luís Ruano, João José Cerqueira

**Affiliations:** 1 Life and Health Sciences Research Institute (ICVS), School of Medicine, Campus de Gualtar, University of Minho, 4710-057 Braga, Portugal; 2 Neuroradiology Department, Hospital de Braga, Rua da Comunidades Lusíadas 133, Braga, Portugal; 3 Neurology Department, Centro Hospitalar de Entre Douro e Vouga, Rua Dr. Cândido Pinho 5, 4520-211 Santa Maria da Feira, Portugal; 4 EPIUnit, Institute of Public Health, University of Porto, Rua das Taipas 135, 4050-600 Porto, Portugal; 5 Neurology Department, Hospital de Braga, Rua da Comunidades Lusíadas 133, Braga, Portugal

**Keywords:** Brain volume changes, Cognition, Multiple sclerosis

## Abstract

**Background:**

The most reliable magnetic resonance imaging (MRI) marker of cognitive dysfunction in multiple sclerosis (MS) is brain atrophy. However, 1-year volumetric changes prior to cognitive assessment were never studied as potential predictors of cognition, which we aim to assess with this pilot work.

**Methods:**

Twenty-two MS patients were submitted to a baseline measure of 83 regional brain volumes with MRI and re-evaluated 1 year later; they were also tested with the Brief International Cognitive Assessment for MS (BICAMS): sustained attention and processing speed were examined with the Symbol Digit Modalities Test (SDMT), verbal and visuo-spatial learning and memory with the learning trials from the California Verbal Learning Test-II (CVLT) and the Brief Visuo-spatial Memory Test-revised (BVMT), respectively. Controlling for age, sex, and years of education, a multivariate linear regression model was created for each cognitive score at 1-year follow-up in a backward elimination manner, considering cross-sectional regional volumes and 1-year volume changes as potential predictors.

**Results:**

Decreases in the volumes of the left amygdala and the right lateral orbitofrontal cortex in the year prior to assessment were identified as possible predictors of worse performance in verbal memory (*P* = 0.009) and visuo-spatial memory (*P* = 0.001), respectively, independently of cross-sectional brain regional volumes at time of testing.

**Conclusion:**

Our work reveals novel 1-year regional brain volume changes as potential predictors of cognitive deficits in MS. This suggests a possible role of these regions in such deficits and might contribute to uncover cognitively deteriorating patients, whose detection is still unsatisfying in clinical practice.

**Supplementary Information:**

The online version contains supplementary material available at 10.1007/s11845-023-03528-x.

## Introduction

Multiple sclerosis (MS) is a chronic demyelinating disorder of the central nervous system with a wide range of motor and sensory symptoms [1]. It leads to working inability, socioeconomic burden, reduced quality of life, and life expectancy [2]. Importantly, this disabling disease affects 2.3 million people worldwide, representing the most common cause of nontraumatic disability in young adults [3]. Although MS is usually recognized by typical presentations that include visual loss, diplopia, ataxia, weakness, sensory disturbances, or urge incontinence [1], cognitive impairment is also now recognized as a common feature of the disease, with an estimated prevalence of 40–70% [4], including deficits on processing speed, attention, learning, and memory as frequently affected domains [5]. Importantly, cognitive dysfunction decreases adherence to treatment and further impacts quality of life of patients with increased unemployment and rates of divorces [2, 4]. However, the underpinnings of these cognitive deficits are still largely unknown and health professionals are poor at detecting them in routine clinical consultation [6].

Additionally, even though the cardinal features of MS lesions are demyelination and gliosis of white matter, the paradigm of the disease has been redefined to one that is also characterized by grey matter damage and widespread neurodegeneration, reflected into brain atrophy [7]. Remarkably, brain atrophy and cognitive impairment are both detectable from the earliest stages of MS [8, 9] and demyelinating lesions play a minor role in comparison with atrophy as correlates of cognitive impairment [10, 11]. Furthermore, among magnetic resonance imaging (MRI) measures investigated as correlates of cognitive dysfunction, grey matter atrophy stands out as the most reliable marker of cognitive function [12]. Therefore, identification of brain volumetric biomarkers of cognitive (dys)function might significantly contribute to a better understanding of this MS feature and a more thorough management of these patients.

Multiple studies have found cross-sectional associations between cognitive function and volume of different brain subregions [10, 13–18], others have correlated variation of cognitive function with changes in brain subregional volumes [19–24] or have predicted cognitive progression based on baseline volume measures [20, 22, 25, 26]. However, how subregional brain volume changes prior to cognitive assessment predict performance has not been assessed in MS. Furthermore, even though yearly brain structural MRI scan is recommended while patients are on disease-modifying treatments [27], to the best of our knowledge, 1-year volumetric changes prior to clinical assessment were never studied as potential predictors of the cognitive state of patients. Hence, in this pilot study, we aimed to evaluate if 1-year volume changes of various brain subregions in structural MRI can potentially predict cognitive scores of an internationally used battery of tests recently validated for the Portuguese population [28], which evaluates attention, processing speed, verbal and visuo-spatial learning and memory. To do so, a cohort of multiple sclerosis patients was submitted to a brain MRI scan and repeated it together with cognitive evaluation after 1 year. Controlling for age, sex, and years of education, we investigated if any 1-year brain volumetric change could predict cognitive scores and created a multiple linear regression model for each score that include 1-year volume changes and cross-sectional volume variables as predictors.

## Materials and methods

### Study design and participants

This study is part of an ongoing longitudinal study (ReCogMS) and was approved by the Ethics Committee of Hospital de Braga (Portugal). The study was conducted in accordance with the principles expressed in the Declaration of Helsinki.

Participants were diagnosed, according to the 2010 McDonald criteria [29], with a relapsing-remitting form of MS [30], showed an Expanded Disability Status Scale rate equal or lower than 4.5 [31] and were at least 18 years old (inclusion criteria). Exclusion criteria were clinical history of other neurological disorder, presence of a major psychiatric disorder, history of learning disability, history of serious head trauma, presence of alcohol or drugs abuse, relapse or steroids treatment within 4 weeks preceding neuropsychological assessment, and any contraindication to MRI. On total, 28 patients were enrolled.

After informed consent, those patients were submitted to a baseline evaluation that included reporting of their age, sex, years of education, time since first MS symptom (which was similar to time since diagnosis), Beck Depression Inventory (BDI), Modified Fatigue Impact Scale (MFIS), Expanded Disability Status Scale (EDSS), MRI, and cognitive evaluation. One year later, patients were re-evaluated with BDI, MFIS, EDSS, MRI, and cognitive assessment. For the latter evaluation, data was collected only from 22 subjects, due to loss of follow-up. During the 12 months of study, patients went through their usual clinical follow-up and any relevant clinical event was registered to ensure that no relapse or steroids treatment occurred within 4 weeks prior to the follow-up cognitive and MRI assessment.

### MRI data acquisition

All MRI assessments were performed at Hospital de Braga (Braga, Portugal) on a clinical approved Siemens Magnetom Avanto 1.5T MRI scanner (Siemens Medical Solutions, Erlangen, Germany) with a 12-channel receive-only head-coil. The imaging protocol included several different acquisitions. For the present study, only the structural acquisitions were considered. For this, a T1-weighted magnetization prepared rapid gradient echo (MPRAGE) sequence was acquired with the following parameters: 176 sagittal slices, TR/TE = 2730/3.48 ms, FA = 7°, slice thickness = 1 mm, slice gap = 0 mm, voxel size = 1 × 1 mm^2^, FoV = 256 mm. Additionally, a T2-weighted sequence was obtained with the following parameters: 60 axial slices, TR/TE = 5750/83 ms, FA = 180°, slice thickness = 3 mm, slice gap = 3mm, voxel size = 1 × 1 mm^2^, FoV = 256 mm.

### MRI data pre-processing

MRI data was processed using the standard semi-automatic workflow implemented in FreeSurfer toolkit version 6.0 (http://surfer.nmr.mgh.harvard.edu/). In summary, the entire pipeline involves 31 processing steps which include the spatial normalization to Talairach standard space, skull stripping, intensity normalization, tessellation of gray matter-white matter boundary, and cortical, subcortical, and WM segmentation. This pipeline has been validated against manual segmentations [32] and is considered reliable across sessions, scanner platforms, updates, and field strengths [33]. It has suffered several improvements throughout the years and details of the procedures are described in several publications [32, 34, 35]. T2-weighted acquisition was used to improve pial surfaces reconstruction. To extract reliable volume estimates for longitudinal data, images were automatically processed with the longitudinal stream [36] implemented in FreeSurfer. Specifically, an unbiased within-subject template space and image [37] is created using robust, inverse consistent registration [38]. Several processing steps, such as skull stripping, Talairach transforms, atlas registration as well as spherical surface maps and parcellations are then initialized with common information from the within-subject template, significantly increasing reliability and statistical power [36]. For the present study, subcortical and cortical volumes were considered according to the Desikan atlas [34], in a total of 83 areas (Supplementary material).

### Cognitive assessment

Participants underwent the Brief International Cognitive Assessment for Multiple Sclerosis (BICAMS), recently validated for the Portuguese population [28], which included the oral version of the Symbol Digit Modalities Test (SDMT), the learning trials from the California Verbal Learning Test-II (CVLT) and the Brief Visuo-spatial Memory Test-revised learning trials (BVMT), as described by Sousa et al.

Briefly, the SDMT examines sustained attention, concentration, and processing speed. The participants were first presented with a series of nine meaningless geometric symbols that were labelled from 1 to 9. Then, during 90 s, they were presented with series of the same symbols (this time not labelled) and asked to say the associated number as rapidly as possible. The test score corresponds to the number of correct responses.

The CVLT is a measure of verbal learning and memory. The test begins with the examiner reading a list of 16 words to the patients, who are then asked to recall as many items as possible in any order. After recall is recorded, the entire list is read again followed by a second attempt. Altogether, this test consists of five of these learning trials. The corresponding outcome is the total number of recalled items over the five trials.

The BVMT assesses visuo-spatial learning and memory. The participant is exposed to a matrix of six abstract geometric designs for 10 s, followed by an unaided recall (form number 1 of the original test was used [39]). Subjects are asked to draw a reproduction of the designs, taking as much time as needed. The scoring criterion was based on location and accuracy of each design (from 0 to 2, maximum total score for each array 12). The outcome consisted on the sum of scores across three trials.

We used the cutoffs with the highest positive predictive value to detect cognitive impairment in MS as defined by Artemiadis et al. [40] Failure on those tests (cognitive impairment) was defined as < 49, < 53, and < 22 for SMDT, CVLT, and BVMT, respectively.

### Statistical analysis

Age, sex, and years of education were treated as potential confounders and so considered as covariates in all multiple linear regression analyses described below. Additionally, total intracranial volume was also included as a covariate in all regression models to account for individual variability of head size.

A 1-year volumetric change score was calculated for each of the 83 brain regions considered and independently for each subject by subtracting volume measured in initial MRI from final volume. For each cognitive score after the 1-year follow-up as the dependent variable, we aimed to create a multivariate regression model considering cross-sectional subregional volumes and 1-year volume changes as covariates, adjusting for the potential confounders. First, we began by performing an univariate screening analysis of each volumetric covariate as a potential predictor of each cognitive score (importantly, although called “univariate” for easier distinction from the definitive multivariate model described below, such analyses were always adjusted for the aforementioned potential confounders and total intracranial volume). Variables whose *β* coefficient *P* value was lower than 0.05 in univariate analysis were considered for the multivariate model, which was created in a backward elimination manner: volumetric covariates with the highest *P* value were individually removed until all brain volume variables (1-year changes or cross-sectional) had a *β* coefficient *P* value lower than 0.05. The coefficient of determination *R*^2^ adjusted for number of predictors (*R*^2^_a_) and root of mean square error (RMSE) were calculated for the multivariate models and relevance of 1-year volumetric changes was examined by the impact on *R*^2^_a_ and RMSE upon removal of those variables.

Additionally, to graph the influence of 1-year volume change measures on each cognitive score, we calculated residuals adjusting for confounders, estimated total intracranial volume, and the cross-sectional volume variables included in the multivariate model. Then, those residuals were predicted by the 1-year volume changes included in the multivariate model using simple linear regression (*β* coefficient *P* value considered significant if lower than 0.05). Equations of those regressions were graphically represented together with individual values.

Volumetric measures were used in mm^3^, age in years, and categorical variable “sex” was coded as 1 for women, 0 for men. Stata software was used to analyze data and generate graphs (StataCorp. 2019. *Stata Statistical Software: Release 16*. College Station, TX: StataCorp LLC).

## Results

From the group of 28 MS patients initially enrolled in this study for baseline MRI scan and cognitive assessment, 22 were re-assessed after 12 months. Those patients are characterized as displayed in Table 1—9 were treated with interferon beta-1a, 6 with interferon beta-1b, 4 with glatiramer acetate, and 3 with natalizumab. At baseline, 6, 11, and 7 patients showed cognitive impairment in SDMT, CVLT, and BVMT, respectively; in the cognitive reevaluation after 1 year, 8, 14, and 3 patients demonstrated cognitive impairment in SDMT, CVLT, and BVMT, respectively.

**Table 1:** Characteristics of MS participants

**Variable**		**Min.**	**Max.**
Age at baseline, years (mean ± SD)	36.863 ± 8.850	21	53
Sex (female % (***N***)**)**	50% (11)	-	-
Education, years (mean ± SD)	11.455 ± 2.972	4	17
Time since 1**st** symptom at baseline, years (mean ± SD)	4.773 ± 3.518	1	13
EDSS score at baseline (median (IQR))	1 (IQR = 2)	-	-
EDSS score after 1-year follow-up (median (IQR))	1.5 (IQR = 1)	-	-
SMDT score at baseline (mean ± SD)	50.636 ± 10.303	31	71
SMDT score after 1-year follow-up (mean ± SD)	51.818 ± 13.280	25	80
CVLT score at baseline (mean ± SD)	51.000 ± 9.842	26	66
CVLT score after 1-year follow-up (mean ± SD])	48.727 ± 8.913	26	64
BVMT score at baseline (mean ± SD)	25.227 ± 7.788	11	34
BVMT score after 1-year follow-up (mean ± SD)	26.727 ± 7.337	4	36

*SD* standard deviation, *N* number of patients, *IQR* interquartile range

Mean change over 1 year of each regional brain volume is presented in Supplementary material. Results of the univariate analyses of cross-sectional regional brain volumes as predictors of performance in each of the three cognitive tests in which *P* > 0.05 are also presented in Supplementary material.

Results of the multivariate regression analyses to determine predictors of performance in each of the cognitive tasks used (SDMT, CVLT, and BVMT) after the 1-year follow-up are presented as follows.

In the univariate analyses, only cross-sectional volumes of the left precentral gyrus, right pericalcarine cortex, and left nucleus accumbens were potentially predictors of SDMT score, when controlling for age, sex, and years of education. Whereas, the first two showed a positive association with this score, the right pericalcarine cortex volume showed a negative *β* coefficient. When combined in a multiple linear regression model adjusting for the same confounders, only the right pericalcarine cortex volume was found to have a significant association with SDMT (Table 2). This model explained approximately 42.5% of the variability among the observed SDMT scores (*R*^2^_a_), with a standard deviation of the residuals of 10.066 (RMSE).

**Table 2:** Subregional brain volume predictors of SDMT

		**Univariate analyses (controlling for age, sex, and years of education)**	**Multivariate analysis**		
**Variable**		***β* [95% CI]**	***P***	***β* [95% CI]**	***P***
Cross sectional volumes	L precentral gyrus	3.875 (0.001–7.749)	0.050	-	-
	R pericalcarine cortex	− 11.971 (− 23.546 to − 0.396)	0.043	− 11.971 (− 23.546 to − 0.396)	0.043
	L nucleus accumbens	49.892 (5.660–94.124)	0.029	-	-
Age		#		− 0.559 (− 1.141 to 0.023)	0.059
Sex		#		− 4.175 (− 16.873 to 8.523)	0.496
Education years		#		1.175 (− 0.427 to 2.777)	0.140
Constant		#		59.619 (− 25.028 to 144.267)	0.155
				*R*^2^_a_ = 0.425 RMSE = 10.066	

#: *β* coefficient dependent on the univariate analysis

*L* left, *R* right, *CI* confidence interval

**Fig. 1:**
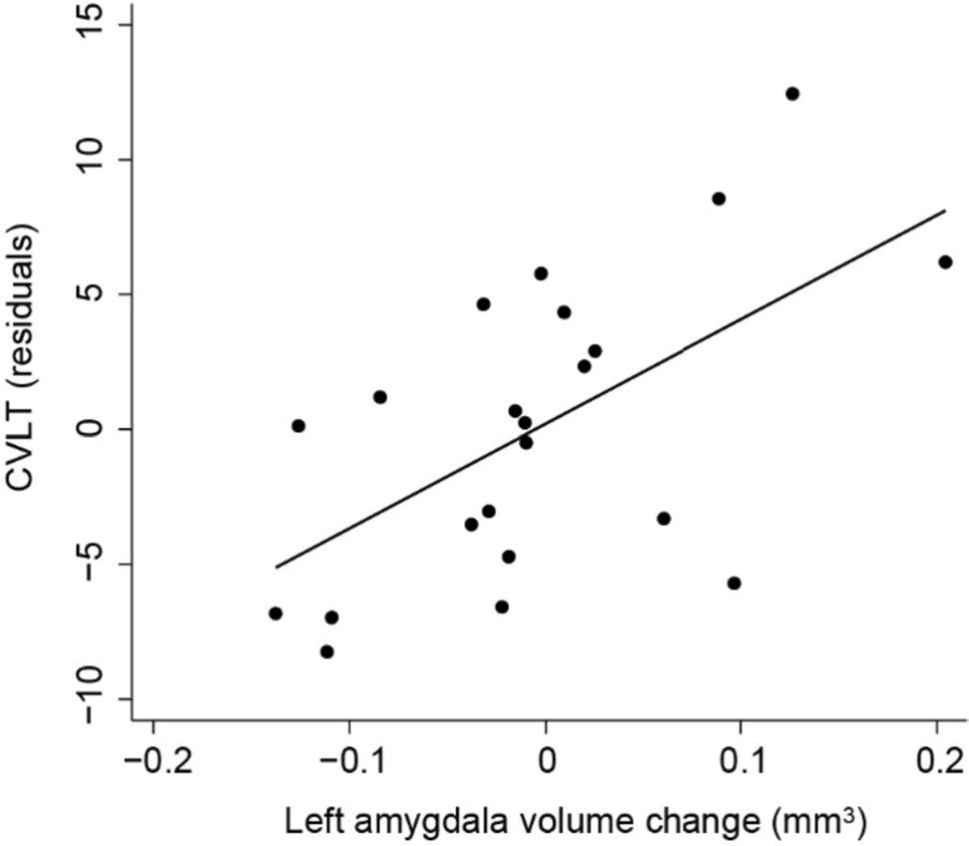
CVLT residuals controlling for age, sex, years of education, estimated total intracranial volume, and brainstem cross-sectional volume predicted by 1-year volume change of left amygdala by the following simple linear regression equation: *Y* = 0.205 + 38.699*x* + *ε* (*β* coefficient 95% confidence interval = [13.854–63.544],* P* = 0.004). Line represents regression model; each dot represents individual values

Regarding verbal memory performance score, in the univariate analyses, cross-sectional volumes of left parahippocampal gyrus, left pericalcarine cortex, and brainstem had a *β* coefficient *P* value lower than 0.05, with the brainstem volume showing a positive association, as opposed the other cross-sectional volume variables. Left amygdala 1-year volume change was also identified as a potential predictor in the univariate analysis. In the multivariate regression analysis, cross-sectional volume of brainstem and volume change of left amygdala were kept in the model (Table 3), which revealed a *R*^2^_a_ and a RMSE of 0.661 and 5.191, respectively. If left amygdala volume change in 1 year was removed from the model, *R*^2^_a_ would drop to 0.489 and RMSE would increase to 6.373. Additionally, age was negatively associated with this score. Finally, adjusting for potential confounders, estimated total intracranial volume and brainstem cross-sectional volume, residuals of CVLT were significantly predicted by left amygdala 1-year volume change: for given age, sex, years of education, estimated total intracranial volume, and brainstem cross-sectional volume, each 0.1 mm^3^ decrease from previous year in volume of left amygdala leads to an average decrease of approximately 3.9 points in CVLT score (Fig. 1).

**Table 3:** Subregional brain volume predictors of CVLT

		**Univariate analyses (controlling for age, sex, and years of education)**	**Multivariate analysis**		
**Variable**		***β* [95% CI]**	***P***	***β* [95% CI]**	***P***
Cross sectional volumes	L parahippocampal gyrus	− 10.209 (− 18.432 to − 1.985)	0.018	-	-
	L pericalcarine cortex	− 8.767 (− 15.651 to − 1.883)	0.016	-	-
	Brainstem	3.947 (0.955–6.939)	0.013	3.784 (1.331–6.237)	0.005
1-year volume changes	L amygdala	44.502 (6.788–82.216)	0.024	42.334 (12.444–72.224)	0.009
Age		#		− 0.649 (− 0.938 to − 0.359)	< 0.001
Sex		#		− 0.581 (− 7.089 to 5.927)	0.852
Education years		#		0.174 (− 0.719 to 1.067)	0.684
Constant		#		86.470 (44.028–128.912)	0.001
				*R*^2^_a_ = 0.661 RMSE = 5.191	

#: *β* coefficient dependent on the univariate analysis

*L* left, *R* right, *CI* confidence interval

**Fig. 2:**
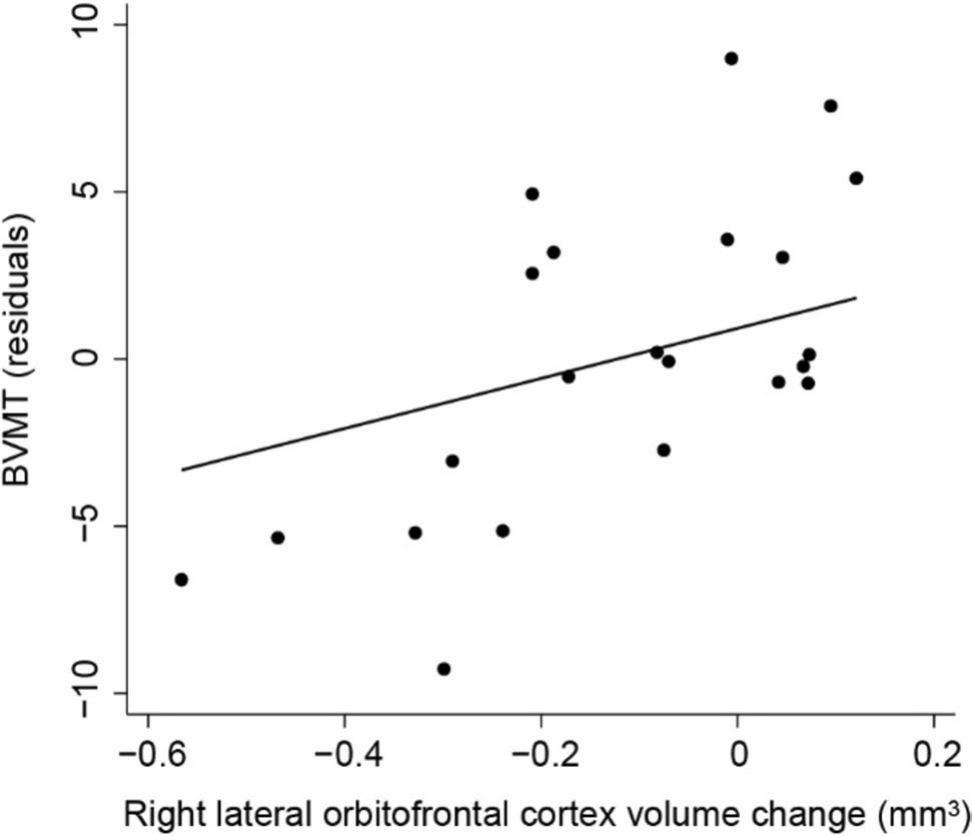
BVMT residuals adjusting for age, sex, years of education, estimated total intracranial volume, and cross-sectional volume of left entorhinal cortex predicted by 1-year volume change of right lateral orbitofrontal cortex by the following simple linear regression equation: *Y* = 1.982 + 16.185*x* + *ε* (*β* coefficient 95% confidence interval = [7.534–24.837];* P* = 0.001). Line represents regression model; each dot represents individual values

Cross-sectional volumes of left entorhinal cortex, left medial orbitofrontal cortex, left parahippocampal gyrus, right frontal pole and 4th ventricle, and 1-year volume changes of right lateral orbitofrontal cortex and left caudate nucleus were found to be significantly associated with visuo-spatial memory when tested individually—whereas the 1-year volume change measures and 4th ventricle cross-sectional volume showed positive associations, the others revealed negative *β* coefficients. When performing the multivariate analysis, cross-sectional volume of left entorhinal cortex and volume change of right lateral orbitofrontal cortex were kept as significant independent variables of BVMT score (Table 4). *R*^2^_a_ would drop from 0.738 to 0.466 and RMSE would increase from 3.755 to 5.364 if volume change of right lateral orbitofrontal cortex was removed from final model. Age was negatively associated with the outcome, with women patients showing significantly lower scores. Controlling for confounders, estimated total intracranial volume and cross-sectional volume of left entorhinal cortex, BVMT residuals were significantly predicted by 1-year volume change of right lateral orbitofrontal cortex: each 0.1 mm^3^ decrease from 1 year ago in volume of right lateral orbitofrontal cortex leads to reduction of approximately 1.6 points on average in BVMT, for given age, sex, years of education, estimated total intracranial volume, and cross-sectional volume of left entorhinal cortex (Fig. 2).

**Table 4:** Subregional brain volume predictors of BVMT

		**Univariate analyses (controlling for age, sex, and years of education)**	**Multivariate analysis**		
**Variable**		***β* [95% CI]**	***P***	***β* [95% CI]**	***P***
Cross sectional volumes	L entorhinal cortex	− 7.691 (− 14.928 to − 0.453)	0.039	− 8.560 (− 13.673 to − 3.447)	0.003
	L medial orbitofrontal cortex	− 5.973 (− 10.286 to − 1.661)	0.010		
	L parahippocampal gyrus	− 7.807 (− 14.410 to − 1.204)	0.023	-	-
	R frontal pole	− 17.477 (− 34.473 to − 0.482)	0.045	-	-
	4th ventricle	6.650 (0.226–13.073)	0.043	-	-
1-year volume changes	R lateral orbitofrontal cortex	18.758 (5.362–32.154)	0.009	20.241 (9.972–30.510)	0.001
	L caudate nucleus	25.517 (1.820–49.627)	0.036	-	-
Age		#		− 0.319 (− 0.526 to − 0.112)	0.005
Sex		#		− 7.305 (− 13.470 to − 1.141)	0.023
Education years		#		0.597 (− 0.070 to 1.264)	0.076
Constant		#		43.804 (8.939–78.669)	0.017
				*R*^2^_a_ = 0.738 RMSE = 3.755	

#: *β* coefficient dependent on the univariate analysis

*L* left, *R* right, *CI* confidence interval

## Conclusions and discussion

Our study has shown that volume subregional changes in the year before cognitive assessment may contribute to predict performance in learning and memory in MS patients. In particular, volume decreases in left amygdala were associated with worse verbal learning and memory, whereas reduction in right lateral orbitofrontal cortex volumes was associated with inferior performance in visuo-spatial learning and memory.

The amygdala has long been associated with learning and memory in interaction with the hippocampus and other regions [41, 42]. This study strengthens and might extend previous findings that showed a cross-sectional association between reduced amygdala volume and performance in CVLT test [14, 43], by highlighting that, independently of the cross-sectional volume, an atrophy of the left amygdala in the year prior to the assessment is a potential predictor of worse performance in this verbal memory test. It is important to note that, in our study, only an atrophy of the left, but not the right, amygdala, was associated with verbal memory performance. This is in light with the fact that subjects with physical damage of the left amygdala are disproportionately impaired on memory for narratives as compared with memory for pictures [44] and supports a lateralized involvement of left amygdala in verbal over nonverbal memory that has already been described in other neuropsychiatric disorders [45].

Our finding that atrophy of the right orbitofrontal cortex in the year prior to assessment is a possible predictor of worse performance in visuospatial memory performance extends previous findings associating cross-sectional volume of the orbitofrontal cortex with memory performance in MS [46]. Neural activity impairment in orbitofrontal cortex and neurodegenerative changes in its circuitry have also been reported in other neurological conditions characterized by memory impairment, including in Alzheimer’s disease [47]. Interestingly, the orbitofrontal cortex is suggested to play a modulatory role over amygdala activity in learning and memory [48, 49]. However, whereas we found an association between verbal memory function and left amygdala atrophy, our analyses revealed an association between visuospatial memory and atrophy of the right lateral orbitofrontal cortex. This is not surprising, and is consistent with a solid body of evidence associating visuospatial functioning to the right hemisphere and verbal functioning to the left, including in learning and memory processing [50].

Some caveats must be considered in this study. Firstly, as six participants were not re-evaluated with MRI and submitted to cognitive assessment due to loss of follow-up, some selection bias might have been introduced. Secondly, although we adjusted our analyses to well established potential confounders, as an observational study, others might have been neglected. These findings are also not necessarily specific to MS, as healthy controls were not compared to our sample. Furthermore, although our results match previous literature in associating learning and memory functioning to amygdala and orbitofrontal cortex, these do not imply causation but may rather reflect a neural adaptative mechanism in response to a primary damage. Finally, although our novel findings associating prior volume changes to cognitive function are inferred from statistical analyses controlling for potential confounders and cross-sectional volume measures, additional evidence is necessary for robust application in clinical practice. In particular, future studies should be powered with bigger sample size (such as to tolerate multiple comparisons correction, analyses of the various possible interactions, and integration of several covariates with lower risk of “overfitting”) or designed in a hypothesis-driven manner specifically directed for volume changes of left amygdala and right lateral orbitofrontal cortex.

Our work suggests that other measures of brain regional volumes should be taken into account when considering MRI as a potentially powerful tool to predictive cognition in MS. Such dysfunction is still poorly detected although highly impactful [2, 4, 6].

### Supplementary Information

Below is the link to the electronic supplementary material.

Supplementary file1 (DOCX 106 KB)

## Data Availability

The data that support the findings of this study are available within the paper and its supplementary information file. Raw data that support the findings of this study are available from the corresponding author upon request.
