# Supplementary material for: One-year regional brain volume changes as potential predictors of cognitive function in multiple sclerosis: a pilot study
Source: Ir J Med Sci. 2023 Sep 29;193(2):957–65. doi: 10.1007/s11845-023-03528-x (PMC10961282; doi:10.1007/s11845-023-03528-x)
Supplement: Supplementary file 1 — Supplementary file1 (DOCX 106 KB) [file 11845_2023_3528_MOESM1_ESM.docx]

Table 1. One-year percent change of subregional brain volume measures.

|  | Sectional volume variable | One-year average volume change (%) | Standard deviation |
| --- | --- | --- | --- |
|  | L caudal anterior cingulate cortex | -2.391 | 5.250 |
|  | L caudal middle frontal gyrus | -1.801 | 2.980 |
|  | L entorhinal cortex | -1.582 | 3.710 |
|  | L fusiform gyrus | -1.121 | 2.837 |
|  | L inferior parietal lobule | -1.771 | 1.911 |
|  | L inferior temporal gyrus | -1.943 | 3.121 |
|  | L isthmus cingulate cortex | 1.614 | 3.359 |
|  | L lateral occipital cortex | -2.260 | 4.233 |
|  | L lateral orbitofrontal cortex | -1.613 | 3.510 |
|  | L lingual gyrus | -1.529 | 2.061 |
|  | L medial orbitofrontal cortex | -0.643 | 2.614 |
|  | L middle temporal gyrus | -1.619 | 2.577 |
|  | L parahippocampal gyrus | 0.472 | 4.202 |
|  | L paracentral lobule | -0.743 | 3.193 |
|  | L pars opercularis | -0.094 | 4.216 |
|  | L pars orbitalis | -2.354 | 4.222 |
|  | L pars triangularis | -1.515 | 3.619 |
|  | L pericalcarine cortex | -2.640 | 4.690 |
|  | L postcentral gyrus | -1.368 | 2.764 |
|  | L posterior cingulate cortex | -0.292 | 2.241 |
|  | L precentral gyrus | -0.990 | 2.924 |
|  | L precuneus cortex | -0.659 | 2.386 |
|  | L rostral anterior cingulate cortex | -1.786 | 3.840 |
|  | L rostral middle cingulate cortex | -1.098 | 2.037 |
|  | L superior frontal gyrus | -1.050 | 1.886 |
|  | L superior parietal lobule | -1.928 | 5.484 |
|  | L superior temporal gyrus | -1.530 | 3.102 |
|  | L supramarginal gyrus | -1.403 | 2.221 |
|  | L frontal pole | -2.404 | 3.665 |
|  | L temporal pole | 0.709 | 5.216 |
|  | L insula | -2.172 | 4.551 |
|  | R caudal anterior cingulate cortex | -0.057 | 4.944 |
|  | R caudal middle frontal gyrus | -1.295 | 2.577 |
|  | R entorhinal cortex | 0.203 | 10.150 |
|  | R fusiform gyrus | -0.135 | 1.944 |
|  | R inferior parietal lobule | -0.644 | 3.150 |
|  | R inferior temporal gyrus | -1.674 | 3.633 |
|  | R isthmus cingulate cortex | 0.751 | 5.040 |
|  | R lateral occipital cortex | -2.304 | 6.236 |
|  | R lateral orbitofrontal cortex | -1.536 | 2.457 |
|  | R lingual gyrus | -0.956 | 1.967 |
|  | R medial orbitofrontal cortex | -0.738 | 3.229 |
|  | R middle temporal gyrus | -1.164 | 1.923 |
|  | R parahippocampal gyrus | -0.688 | 3.089 |
|  | R paracentral lobule | -0.458 | 4.087 |
|  | R pars opercularis | -1.212 | 2.341 |
|  | R pars orbitalis | -3.690 | 7.696 |
|  | R pars triangularis | -1.694 | 3.498 |
|  | R pericalcarine cortex | -1.417 | 6.775 |
|  | R postcentral gyrus | -0.656 | 2.883 |
|  | R posterior cingulate cortex | 0.120 | 4.872 |
|  | R precentral gyrus | -0.124 | 2.694 |
|  | R precuneus cortex | -0.500 | 2.624 |
|  | R rostral anterior cingulate cortex | -0.831 | 3.134 |
|  | R rostral middle cingulate cortex | -1.302 | 1.847 |
|  | R superior frontal gyrus | -1.292 | 1.764 |
|  | R superior parietal lobule | -1.355 | 3.579 |
|  | R superior temporal gyrus | -0.655 | 1.866 |
|  | R supramarginal gyrus | -0.729 | 2.002 |
|  | R frontal pole | -2.185 | 4.030 |
|  | R temporal pole | -2.937 | 5.411 |
|  | R insula | -2.995 | 7.582 |
|  | L lateral ventricle | 2.544 | 13.684 |
|  | L cerebellum cortex | -0.753 | 1.416 |
|  | L thalamus | -1.601 | 2.784 |
|  | L caudate nucleus | -2.382 | 2.843 |
|  | L putamen | -1.986 | 2.683 |
|  | L pallidum | -0.632 | 4.265 |
|  | Third ventricle | 0.087 | 10.176 |
|  | Fourth ventricle | 2.029 | 4.244 |
|  | Brainstem | -0.722 | 1.788 |
|  | L hippocampus | -2.081 | 4.257 |
|  | L amygdala | -0.137 | 6.405 |
|  | L nucleus accumbens | -0.292 | 5.826 |
|  | R lateral ventricle | 2.442 | 13.909 |
|  | R cerebellum cortex | -0.678 | 1.662 |
|  | R thalamus | -1.124 | 2.437 |
|  | R caudate nucleus | -1.992 | 3.796 |
|  | R putamen | -2.132 | 4.178 |
|  | R pallidum | -0.676 | 5.520 |
|  | R hippocampus | -1.033 | 2.620 |
|  | R amygdala | -1.566 | 4.498 |
|  | R nucleus accumbens | -1.458 | 9.662 |

Table 2. Subregional brain volume variables as predictors of SDMT.

|  |  | Univariate analyses (controlling for age, sex and years of education) |  |
| --- | --- | --- | --- |
| Variable |  | ***β* [95% CI]** | ***P*** |
| Cross sectional volumes | L caudal anterior cingulate cortex | 5.573 [-6.627 – 17.774] | 0.347 |
|  | L caudal middle frontal gyrus | -1.993 [-9.835 – 5.850] | 0.598 |
|  | L entorhinal cortex | -12.974 [-26.854 – 0.905] | 0.065 |
|  | L fusiform gyrus | 1.183 [-4.054 – 6.419] | 0.639 |
|  | L inferior parietal lobule | 2.538 [-1.482 – 6.557] | 0.199 |
|  | L inferior temporal gyrus | 0.123 [-4.815 – 5.061] | 0.959 |
|  | L isthmus cingulate cortex | -14.476 [-43.697 – 14.745] | 0.309 |
|  | L lateral occipital cortex | 1.509 [-3.378 – 6.397] | 0.522 |
|  | L lateral orbitofrontal cortex | -4.592 [-15.361 – 6.177] | 0.379 |
|  | L lingual gyrus | -6.602 [-13.235 – 0.030] | 0.051 |
|  | L medial orbitofrontal cortex | -4.268 [-13.984 – 5.448] | 0.366 |
|  | L middle temporal gyrus | -0.517 [-4.973 – 3.938] | 0.809 |
|  | L parahippocampal gyrus | -9.949 [23.489 – 3.592] | 0.139 |
|  | L paracentral lobule | -5.794 [-23.721 – 12.134] | 0.503 |
|  | L pars opercularis | -4.404 [-14.891 – 6.083] | 0.387 |
|  | L pars orbitalis | 10.086 [-13.306 – 33.478] | 0.374 |
|  | L pars triangularis | -4.589 [-16.254 – 7.076] | 0.417 |
|  | L pericalcarine cortex | -10.190 [-21.197 – 0.816] | 0.067 |
|  | L postcentral gyrus | 1.027 [-5.670 – 7.725] | 0.749 |
|  | L posterior cingulate cortex | 4.912 [-9.109 – 18.934] | 0.468 |
|  | L precentral gyrus | 3.875 [0.001 – 7.749] | 0.050 |
|  | L precuneus cortex | -0.465 [-6.839 – 5.909] | 0.879 |
|  | L rostral anterior cingulate cortex | 3.332 [-7.681 – 14.346] | 0.530 |
|  | L rostral middle cingulate cortex | 1.224 [-2.026 – 4.475] | 0.436 |
|  | L superior frontal gyrus | 0.384 [-2.825 – 3.594] | 0.803 |
|  | L superior parietal lobule | 0.932 [-4.359 – 6.223] | 0.714 |
|  | L superior temporal gyrus | -0.627 [-4.498 – 3.244] | 0.736 |
|  | L supramarginal gyrus | -2.743 [-5.545 – 0.058] | 0.054 |
|  | L frontal pole | -3.340 [-29.801 – 23.121] | 0.792 |
|  | L temporal pole | -3.208 [-12.065 – 5.649] | 0.454 |
|  | L insula | -0.161 [-10.578 – 10.257] | 0.974 |
|  | R caudal anterior cingulate cortex | 0.106 [-14.879 – 15.091] | 0.988 |
|  | R caudal middle frontal gyrus | -1.011 [-10.411 – 8.389] | 0.823 |
|  | R entorhinal cortex | -1.992 [-9.320 – 5.335] | 0.572 |
|  | R fusiform gyrus | 0.334 [-4.487 – 5.155] | 0.885 |
|  | R inferior parietal lobule | -1.010 [-4.124 – 2.104] | 0.502 |
|  | R inferior temporal gyrus | -2.641 [-6.576 – 1.295] | 0.174 |
|  | R isthmus cingulate cortex | -4.011 [-26.365 – 18.344] | 0.709 |
|  | R lateral occipital cortex | 1.046 [-3.952 – 6.044] | 0.663 |
|  | R lateral orbitofrontal cortex | -4.372 [-13.543 – 4.799] | 0.327 |
|  | R lingual gyrus | -1.481 [-7.755 – 4.794] | 0.624 |
|  | R medial orbitofrontal cortex | -2.261 [-14.188 – 9.666] | 0.693 |
|  | R middle temporal gyrus | -0.262 [-4.140 – 3.615] | 0.888 |
|  | R parahippocampal gyrus | -5.150 [-29.432 – 19.131] | 0.659 |
|  | R paracentral lobule | -4.303 [-21.168 – 12.563] | 0.596 |
|  | R pars opercularis | -4.673 [-16.584 – 7.238] | 0.418 |
|  | R pars orbitalis | 1.033 [-13.887 – 15.953] | 0.885 |
|  | R pars triangularis | -1.021 [-8.177 – 6.134] | 0.766 |
|  | R pericalcarine cortex | -11.971 [-23.546 – 0.396] | 0.043 |
|  | R postcentral gyrus | 0.716 [-4.175 – 5.607] | 0.760 |
|  | R posterior cingulate cortex | -2.009 [-14.805 – 10.786] | 0.744 |
|  | R precentral gyrus | 0.705 [-3.581 – 4.991] | 0.732 |
|  | R precuneus cortex | -3.791 [-10.929 – 3.348] | 0.277 |
|  | R rostral anterior cingulate cortex | -1.632 [-17.011 – 13.747] | 0.825 |
|  | R rostral middle cingulate cortex | 1.958 [-2.133 – 6.049] | 0.325 |
|  | R superior frontal gyrus | 0.406 [-2.538 – 3.350] | 0.774 |
|  | R superior parietal lobule | -0.028 [-4.655 – 4.598] | 0.990 |
|  | R superior temporal gyrus | 0.689 [-5.086 – 6.465] | 0.803 |
|  | R supramarginal gyrus | 0.007 [-5.401 – 5.416] | 0.998 |
|  | R frontal pole | -28.225 [-61.074 – 4.624] | 0.087 |
|  | R temporal pole | -8.204 [-22.597 – 6.190] | 0.245 |
|  | R insula | 32.593 [-7.617 – 12.819] | 0.597 |
|  | L lateral ventricle | -0.249 [-1.388 – 0.890] | 0.649 |
|  | L cerebellum cortex | -0.162 [-1.481 – 1.156] | 0.797 |
|  | L thalamus | 4.711 [-3.402 – 12.824] | 0.236 |
|  | L caudate nucleus | 4.279 [-7.623 – 16.181] | 0.457 |
|  | L putamen | -0.504 [-12.586 – 11.578] | 0.931 |
|  | L pallidum | 8.157 [-20.080 – 36.394] | 0.549 |
|  | Third ventricle | 1.063 [-12.854 – 14.979] | 0.873 |
|  | Fourth ventricle | 4.272 [-9.201 – 17.746] | 0.511 |
|  | Brainstem | 1.104 [-4.254 – 6.461] | 0.668 |
|  | L hippocampus | 1.704 [-12.480 – 15.887] | 0.802 |
|  | L amygdala | 2.053 [-30.657 – 34.763] | 0.896 |
|  | L nucleus accumbens | 49.892 [5.660 – 94.124] | 0.029 |
|  | R lateral ventricle | -0.254 [-1.451 – 0.943] | 0.659 |
|  | R cerebellum cortex | -0.076 [-1.363 – 1.210] | 0.901 |
|  | R thalamus | 1.865 [-5.503 – 9.233] | 0.599 |
|  | R caudate nucleus | 4.417 [-6.675 – 15.508] | 0.411 |
|  | R putamen | -0.453 [-10.756 – 9.849] | 0.927 |
|  | R pallidum | -2.705 [-26.488 – 21.079] | 0.813 |
|  | R hippocampus | 2.625 [-24.242 – 29.493] | 0.839 |
|  | R amygdala | -7.066 [-42.698 – 28.567] | 0.680 |
|  | R nucleus accumbens | -5.553 [-64.081 – 52.974] | 0.843 |
| 1-year volume changes | L caudal anterior cingulate cortex | -6.348 [-94.220 – 81.524] | 0.880 |
|  | L caudal middle frontal gyrus | -2.669 [-35.822 – 30.484] | 0.867 |
|  | L entorhinal cortex | -13.515 [-91.547 – 64.518] | 0.718 |
|  | L fusiform gyrus | 15.005 [-10.603 – 40.613] | 0.232 |
|  | L inferior parietal lobule | 2.322 [-29.667 – 34.311] | 0.880 |
|  | L inferior temporal gyrus | 3.580 [-12.801 – 19.960] | 0.649 |
|  | L isthmus cingulate cortex | -2.423 [-70.271 – 65.425] | 0.941 |
|  | L lateral occipital cortex | 0.270 [-8.742 – 9.282] | 0.950 |
|  | L lateral orbitofrontal cortex | 10.427 [-8.498 – 29.352] | 0.260 |
|  | L lingual gyrus | -4.701 [-41.060 – 31.659] | 0.788 |
|  | L medial orbitofrontal cortex | -16.854 [-57.738 – 24.029] | 0.395 |
|  | L middle temporal gyrus | 0.788 [-17.440 – 19.016] | 0.928 |
|  | L parahippocampal gyrus | 2.579 [-66.630 – 71.787] | 0.938 |
|  | L paracentral lobule | -32.891 [-82.973 – 17.190] | 0.183 |
|  | L pars opercularis | 0.273 [-35.462 – 36.009] | 0.987 |
|  | L pars orbitalis | 17.546 [-28.434 – 63.526] | 0.430 |
|  | L pars triangularis | 2.716 [-29.783 – 35.216] | 0.862 |
|  | L pericalcarine cortex | -8.755 [-63.688 – 46.179] | 0.740 |
|  | L postcentral gyrus | -8.591 [-33.186 – 16.005] | 0.470 |
|  | L posterior cingulate cortex | -63.857 [153.228 – 25.313] | 0.148 |
|  | L precentral gyrus | -4.327 [-18.015 – 9.362] | 0.512 |
|  | L precuneus cortex | -13.091 [-39.933 – 13.751] | 0.317 |
|  | L rostral anterior cingulate cortex | 11.878 [-52.003 – 75.760] | 0.699 |
|  | L rostral middle cingulate cortex | 4.458 [-16.096 – 125.012] | 0.652 |
|  | L superior frontal gyrus | -1.225 [-17.087 – 14.637] | 0.872 |
|  | L superior parietal lobule | -0.807 [-7.498 – 5.884] | 0.801 |
|  | L superior temporal gyrus | 1.704 [-11.490 – 14.897] | 0.788 |
|  | L supramarginal gyrus | -13.133 [-45.233 – 18.968] | 0.399 |
|  | L frontal pole | 32.425 [-99.322 – 164.172] | 0.609 |
|  | L temporal pole | 1.946 [-29.728 – 33.620] | 0.898 |
|  | L insula | 5.578 [-8.488 – 19.643] | 0.413 |
|  | R caudal anterior cingulate cortex | -2.359 [-54.550 – 49.831] | 0.925 |
|  | R caudal middle frontal gyrus | -5.636 [-39.419 – 28.147] | 0.728 |
|  | R entorhinal cortex | -6.782 [-24.291 – 10.727] | 0.424 |
|  | R fusiform gyrus | -10.354 [-43.350 – 22.643] | 0.515 |
|  | R inferior parietal lobule | -2.946 [-15.761 – 9.870] | 0.633 |
|  | R inferior temporal gyrus | 0.492 [-14.640 – 15.625] | 0.946 |
|  | R isthmus cingulate cortex | -18.618 [-66.923 – 29.687] | 0.426 |
|  | R lateral occipital cortex | 0.296 [-4.801 – 5.393] | 0.904 |
|  | R lateral orbitofrontal cortex | 17.644 [-12.025 – 47.313] | 0.226 |
|  | R lingual gyrus | -4.769 [-42.633 – 33.096] | 0.793 |
|  | R medial orbitofrontal cortex | 0.942 [-31.127 – 29.244] | 0.948 |
|  | R middle temporal gyrus | 9.629 [-16.875 – 36.133] | 0.452 |
|  | R parahippocampal gyrus | 7.884 [-85.359 – 101.126] | 0.860 |
|  | R paracentral lobule | -8.355 [-37.940 – 21.230] | 0.558 |
|  | R pars opercularis | -2.038 [-68.773 – 64.697] | 0.949 |
|  | R pars orbitalis | 2.584 [-14.379 – 19.547] | 0.751 |
|  | R pars triangularis | 2.344 [-25.899 – 30.588] | 0.863 |
|  | R pericalcarine cortex | -8.100 [-44.737 – 28.536] | 0.646 |
|  | R postcentral gyrus | -4.760 [-27.181 – 17.661] | 0.659 |
|  | R posterior cingulate cortex | -6.215 [-49.124 – 36.694] | 0.763 |
|  | R precentral gyrus | -7.673 [-23.159 – 7.813] | 0.309 |
|  | R precuneus cortex | -6.166 [-28.334 – 16.001] | 0.564 |
|  | R rostral anterior cingulate cortex | 36.808 [-48.615 – 122.231] | 0.375 |
|  | R rostral middle cingulate cortex | 1.223 [-18.669 – 21.115] | 0.898 |
|  | R superior frontal gyrus | 1.889 [-11.256 – 15.034] | 0.765 |
|  | R superior parietal lobule | -1.389 [-13.984 – 11.205] | 0.818 |
|  | R superior temporal gyrus | 11.308 [-16.011 – 38.626] | 0.393 |
|  | R supramarginal gyrus | -4.263 [-36.714 – 28.188] | 0.784 |
|  | R frontal pole | 57.060 [-215.521 – 26.402] | 0.117 |
|  | R temporal pole | 4.198 [-17.380 – 25.775] | 0.686 |
|  | R insula | 0.752 [-6.674 – 8.179] | 0.833 |
|  | L lateral ventricle | 0.097 [-2.582 – 2.776] | 0.940 |
|  | L cerebellum cortex | 2.228 [-6.166 – 10.622] | 0.581 |
|  | L thalamus | 12.689 [-15.128 – 40.507] | 0.348 |
|  | L caudate nucleus | 36.528 [-10.523 – 83.579] | 0.119 |
|  | L putamen | -10.177 [-48.659 – 28.304] | 0.583 |
|  | L pallidum | -16.345 [-99.666 – 66.975] | 0.683 |
|  | Third ventricle | 1.694 [-24.109 – 27.497] | 0.891 |
|  | Fourth ventricle | -10.809 [-84.218 – 62.599] | 0.759 |
|  | Brainstem | 1.883 [-14.243 – 18.009] | 0.808 |
|  | L hippocampus | 4.977 [-28.461 – 38.415] | 0.756 |
|  | L amygdala | 38.801 [-23.559 – 101.161] | 0.206 |
|  | L nucleus accumbens | 77.229 [-103.194 – 257.652] | 0.378 |
|  | R lateral ventricle | 0.129 [-2.690 – 2.948] | 0.924 |
|  | R cerebellum cortex | 1.827 [-5.877 – 9.531] | 0.622 |
|  | R thalamus | 11.717 [-19.926 – 43.361] | 0.444 |
|  | R caudate nucleus | 1.127 [-29.857 – 31.746] | 0.949 |
|  | R putamen | -1.809 [-26.674 – 23.057] | 0.879 |
|  | R pallidum | 20.416 [-16.685 – 75.516] | 0.195 |
|  | R hippocampus | 1.630 [-49.685 – 52.945] | 0.947 |
|  | R amygdala | 47.439 [-21.259 – 116.138] | 0.163 |
|  | R nucleus accumbens | -31.910 [-147.218 – 83.398] | 0.566 |

L, left; R, right; CI, confidence interval

Table 3. Subregional brain volume variables as predictors of CVLT.

|  |  | Univariate analyses (controlling for age, sex and years of education) |  |
| --- | --- | --- | --- |
| Variable |  | ***β* [95% CI]** | ***P*** |
| Cross sectional volumes | L caudal anterior cingulate cortex | 0.511 [-7.988 – 9.010] | 0.900 |
|  | L caudal middle frontal gyrus | -1.107 [-6.435 – 4.222] | 0.666 |
|  | L entorhinal cortex | -4.294 [-14.537 – 5.949] | 0.387 |
|  | L fusiform gyrus | 0.571 [-2.988 – 4.131] | 0.738 |
|  | L inferior parietal lobule | 0.186 [-2.683 – 3.056] | 0.892 |
|  | L inferior temporal gyrus | 0.282 [-3.060 – 3.624] | 0.860 |
|  | L isthmus cingulate cortex | -6.630 [-26.791 – 13.532] | 0.496 |
|  | L lateral occipital cortex | -1.233 [-4.524 – 2.057] | 0.438 |
|  | L lateral orbitofrontal cortex | -2.326 [-9.702 – 5.051] | 0.513 |
|  | L lingual gyrus | -3.653 [-8.350 – 1.043] | 0.119 |
|  | L medial orbitofrontal cortex | -2.076 [-8.744 – 4.591] | 0.519 |
|  | L middle temporal gyrus | -1.754 [-4.632 – 1.124] | 0.215 |
|  | L parahippocampal gyrus | -10.209 [-18.432 – -1.985] | 0.018 |
|  | L paracentral lobule | -5.110 [-17.130 – 6.910] | 0.381 |
|  | L pars opercularis | -2.448 [-9.609 – 4.714] | 0.479 |
|  | L pars orbitalis | 3.861 [-12.265 – 19.986] | 0.619 |
|  | L pars triangularis | 0.405 [-7.664 – 8.474] | 0.917 |
|  | L pericalcarine cortex | -8.767 [-15.651 – -1.883] | 0.016 |
|  | L postcentral gyrus | -1.799 [-6.250 – 2.652] | 0.404 |
|  | L posterior cingulate cortex | -1.694 [-11.313 – 7.925] | 0.714 |
|  | L precentral gyrus | 0.813 [-2.126 – 3.751] | 0.566 |
|  | L precuneus cortex | -0.124 [-4.445 – 4.197] | 0.952 |
|  | L rostral anterior cingulate cortex | -1.867 [-9.358 – 5.624] | 0.605 |
|  | L rostral middle cingulate cortex | 0.384 [-1.852 – 2.620] | 0.721 |
|  | L superior frontal gyrus | -0.832 [-2.965 – 1.302] | 0.421 |
|  | L superior parietal lobule | 0.849 [-2.723 – 4.420] | 0.621 |
|  | L superior temporal gyrus | -0.526 [-3.144 – 2.091] | 0.676 |
|  | L supramarginal gyrus | -1.434 [-3.433 – 0.564] | 0.148 |
|  | L frontal pole | -3.938 [-21.781 – 13.906] | 0.646 |
|  | L temporal pole | -1.853 [-7.883 – 4.177] | 0.524 |
|  | L insula | 0.530 [-6.521 – 7.582] | 0.875 |
|  | R caudal anterior cingulate cortex | -0.965 [-11.103 – 9.174] | 0.843 |
|  | R caudal middle frontal gyrus | -3.120 [-9.280 – 3.040] | 0.299 |
|  | R entorhinal cortex | -1.383 [-6.344 – 3.578] | 0.563 |
|  | R fusiform gyrus | -1.446 [-4.623 – 1.731] | 0.349 |
|  | R inferior parietal lobule | -1.000 [-3.073 – 1.074] | 0.322 |
|  | R inferior temporal gyrus | -0.788 [ -3.587– 2.010] | 0.559 |
|  | R isthmus cingulate cortex | -4.869 [-19.860 – 10.122] | 0.501 |
|  | R lateral occipital cortex | -1.480 [-4.794 – 1.835] | 0.358 |
|  | R lateral orbitofrontal cortex | -3.449 [-9.591 – 2.692] | 0.251 |
|  | R lingual gyrus | -1.704 [-5.891 – 2.483] | 0.401 |
|  | R medial orbitofrontal cortex | -1.511 [-9.592 – 6.569] | 0.697 |
|  | R middle temporal gyrus | -0.665 [-3.269 – 1.940] | 0.596 |
|  | R parahippocampal gyrus | -10.305 [-25.930 – 5.320] | 0.181 |
|  | R paracentral lobule | -4.464 [-15.748 – 6.819] | 0.414 |
|  | R pars opercularis | -3.625 [-11.640 – 4.389] | 0.352 |
|  | R pars orbitalis | 0.829 [-9.275 – 10.933] | 0.864 |
|  | R pars triangularis | -2.851 [-7.472 – 1.769] | 0.209 |
|  | R pericalcarine cortex | -7.743 [-15.688 – 0.201] | 0.055 |
|  | R postcentral gyrus | -0.585 [-3.894 – 2.724] | 0.713 |
|  | R posterior cingulate cortex | -2.567 [-11.158 – 6.024] | 0.535 |
|  | R precentral gyrus | -0.501 [-3.403 – 2.402] | 0.719 |
|  | R precuneus cortex | -2.261 [-7.139 – 2.617] | 0.340 |
|  | R rostral anterior cingulate cortex | -4.946 [-15.045 – 5.154] | 0.315 |
|  | R rostral middle cingulate cortex | 0.634 [-2.206 – 3.473] | 0.643 |
|  | R superior frontal gyrus | -0.471 [-2.455 – 1.512] | 0.621 |
|  | R superior parietal lobule | 0.219 [-2.913 – 3.351] | 0.884 |
|  | R superior temporal gyrus | -0.352 [-4.268 –3.563] | 0.851 |
|  | R supramarginal gyrus | -1.760 [-5.303 – 1.784] | 0.308 |
|  | R frontal pole | -12.744 [-36.244 – 10.755] | 0.267 |
|  | R temporal pole | -5.280 [-15.074 – 4.513] | 0.270 |
|  | R insula | 1.970 [-4.936 – 8.876] | 0.554 |
|  | L lateral ventricle | -0.019 [-0.795 – 0.758] | 0.960 |
|  | L cerebellum cortex | 0.658 [-0.167 – 1.482] | 0.110 |
|  | L thalamus | 2.971 [-2.559 – 8.502] | 0.272 |
|  | L caudate nucleus | 2.022 [-6.116 – 10.159] | 0.606 |
|  | L putamen | -0.182 [-8.368 – 8.004] | 0.963 |
|  | L pallidum | 10.871 [-7.603 – 29.345] | 0.230 |
|  | Third ventricle | 0.656 [-8.772 – 10.084] | 0.885 |
|  | Fourth ventricle | 7.696 [-0.612 – 16.004] | 0.067 |
|  | Brainstem | 3.947 [0.955 – 6.939] | 0.013 |
|  | L hippocampus | 1.675 [-7.912 – 11.261] | 0.716 |
|  | L amygdala | -1.914 [-24.062 – 20.233] | 0.857 |
|  | L nucleus accumbens | 7.921 [-26.735 – 42.577] | 0.635 |
|  | R lateral ventricle | 0.012 [-0.804 – 0.827] | 0.976 |
|  | R cerebellum cortex | 0.551 [-0.270 – 1.373] | 0.174 |
|  | R thalamus | 4.194 [-0.324 – 8.713] | 0.067 |
|  | R caudate nucleus | 3.357 [-4.113 – 10.828] | 0.355 |
|  | R putamen | 1.978 [-4.924 – 8.880] | 0.552 |
|  | R pallidum | 5.302 [-10.593 – 21.196] | 0.490 |
|  | R hippocampus | 1.118 [-17.098 – 19.333] | 0.898 |
|  | R amygdala | -6.028 [-30.087 – 18.032] | 0.603 |
|  | R nucleus accumbens | -9.723 [-49.084 – 29.639] | 0.608 |
| 1-year volume changes | L caudal anterior cingulate cortex | 12.037 [-47.190 – 71.265] | 0.672 |
|  | L caudal middle frontal gyrus | -1.846 [-24.303 – 20.612] | 0.864 |
|  | L entorhinal cortex | -8.293 [-61.193 – 44.608] | 0.744 |
|  | L fusiform gyrus | 13.239 [-3.516 – 29.993] | 0.113 |
|  | L inferior parietal lobule | 6.262 [-15.168 – 27.693] | 0.544 |
|  | L inferior temporal gyrus | 5.095 [-5.744 – 15.934] | 0.334 |
|  | L isthmus cingulate cortex | -25.476 [-69.418 – 18.466] | 0.237 |
|  | L lateral occipital cortex | 1.293 [-4.774 – 7.361] | 0.657 |
|  | L lateral orbitofrontal cortex | 6.552 [-6.344 – 19.448] | 0.297 |
|  | L lingual gyrus | -8.606 [-32.870 – 15.657] | 0.463 |
|  | L medial orbitofrontal cortex | 3.351 [-24.941 – 31.644] | 0.805 |
|  | L middle temporal gyrus | 6.391 [-5.487 – 18.268] | 0.271 |
|  | L parahippocampal gyrus | -7.588 [-54.307 – 39.132] | 0.735 |
|  | L paracentral lobule | -24.805 [-58.236 – 8.625] | 0.135 |
|  | L pars opercularis | -3.051 [-27.205 – 21.103] | 0.792 |
|  | L pars orbitalis | 20.177 [-9.748 – 50.102] | 0.172 |
|  | L pars triangularis | 6.427 [-15.346 – 28.199] | 0.540 |
|  | L pericalcarine cortex | -11.715 [-48.540 – 25.111] | 0.510 |
|  | L postcentral gyrus | -2.761 [-19.642 – 14.120] | 0.733 |
|  | L posterior cingulate cortex | -14.104 [-78.357 – 50.149] | 0.648 |
|  | L precentral gyrus | -0.640 [-10.035 – 8.756] | 0.887 |
|  | L precuneus cortex | -4.654 [-23.273 – 13.964] | 0.603 |
|  | L rostral anterior cingulate cortex | 16.842 [-25.716 – 59.400] | 0.414 |
|  | L rostral middle cingulate cortex | 3.695 [-10.183 – 17.573] | 0.580 |
|  | L superior frontal gyrus | 1.471 [-9.255 – 12.197] | 0.775 |
|  | L superior parietal lobule | 0.725 [-3.801 – 5.251] | 0.739 |
|  | L superior temporal gyrus | 2.598 [-6.255 – 11.450] | 0.543 |
|  | L supramarginal gyrus | 6.786 [-15.171 – 28.744] | 0.522 |
|  | L frontal pole | 37.527 [-50.252 – 125.306] | 0.378 |
|  | L temporal pole | 0.285 [-21.182 – 21.753] | 0.978 |
|  | L insula | 4.387 [-5.068 – 13.842] | 0.340 |
|  | R caudal anterior cingulate cortex | -8.452 [-43.532 – 26.628] | 0.617 |
|  | R caudal middle frontal gyrus | 0.798 [-25.742 – 20.110] | 0.798 |
|  | R entorhinal cortex | -5.267 [-17.049 – 6.515] | 0.357 |
|  | R fusiform gyrus | -12.432 [-34.112 – 9.248] | 0.242 |
|  | R inferior parietal lobule | -2.309 [-10.969 – 6.351] | 0.580 |
|  | R inferior temporal gyrus | 1.207 [-9.026 – 11.440] | 0.806 |
|  | R isthmus cingulate cortex | -13.003 [-45.683 – 19.676] | 0.411 |
|  | R lateral occipital cortex | 0.843 [-2.582 – 4.269] | 0.609 |
|  | R lateral orbitofrontal cortex | 4.752 [-16.170 – 25.674] | 0.637 |
|  | R lingual gyrus | 0.404 [-25.302 – 26.111] | 0.974 |
|  | R medial orbitofrontal cortex | -6.165 [-26.353 – 14.023] | 0.527 |
|  | R middle temporal gyrus | -2.683 [-20.912 – 15.546] | 0.759 |
|  | R parahippocampal gyrus | -5.181 [-68.349 – 57.987] | 0.864 |
|  | R paracentral lobule | 0.005 [-20.259 – 20.270] | 1.000 |
|  | R pars opercularis | -5.098 [-50.230 – 40.035] | 0.814 |
|  | R pars orbitalis | 3.970 [-7.364 – 15.305] | 0.469 |
|  | R pars triangularis | 5.854 [-13.044 – 24.752] | 0.521 |
|  | R pericalcarine cortex | -6.082 [-30.861 – 18.698] | 0.610 |
|  | R postcentral gyrus | -2.434 [-17.664 – 12.796] | 0.739 |
|  | R posterior cingulate cortex | -1.540 [-30.682 – 17.601] | 0.912 |
|  | R precentral gyrus | -7.863 [-17.877 – 2.151] | 0.115 |
|  | R precuneus cortex | -2.900 [-18.001 – 12.201] | 0.689 |
|  | R rostral anterior cingulate cortex | 0.438 [-58.918 – 59.795] | 0.988 |
|  | R rostral middle cingulate cortex | 0.486 [-12.994 – 13.966] | 0.940 |
|  | R superior frontal gyrus | -0.278 [-9.207 – 8.651] | 0.948 |
|  | R superior parietal lobule | -0.684 [-9.222 – 7.855] | 0.867 |
|  | R superior temporal gyrus | 0.178 [-18.768 – 19.123] | 0.984 |
|  | R supramarginal gyrus | -11.240 [-32.456 – 9.975] | 0.278 |
|  | R frontal pole | -26.393 [-113.978 – 61.193] | 0.532 |
|  | R temporal pole | -0.616 [-15.306 – 14.075] | 0.930 |
|  | R insula | 1.329 [-3.660 – 6.317] | 0.580 |
|  | L lateral ventricle | 0.149 [-1.665 – 1.962] | 0.864 |
|  | L cerebellum cortex | 2.045 [-3.594 – 7.684] | 0.453 |
|  | L thalamus | 10.226 [-8.388 – 28.840] | 0.261 |
|  | L caudate nucleus | 29.886 [-0.725 – 60.496] | 0.55 |
|  | L putamen | 1.742 [-24.565 – 28.049] | 0.890 |
|  | L pallidum | 23.917 [-31.396 – 79.231] | 0.373 |
|  | Third ventricle | 4.449 [-12.881 – 21.779] | 0.594 |
|  | Fourth ventricle | -31.801 [-78.747 – 15.145] | 0.170 |
|  | Brainstem | -2.982 [-13.812 – 7.848] | 0.568 |
|  | L hippocampus | 1.483 [-21.226 – 24.191] | 0.892 |
|  | L amygdala | 44.502 [6.788 – 82.216] | 0.024 |
|  | L nucleus accumbens | 16.478 [-108.545 – 142.501] | 0.784 |
|  | R lateral ventricle | 0.199 [-1.708 – 2.106] | 0.828 |
|  | R cerebellum cortex | -0.299 [-5.556 – 4.958] | 0.905 |
|  | R thalamus | 8.058 [-13.365 – 29.481] | 0.437 |
|  | R caudate nucleus | 5.369 [-15.305 – 26.042] | 0.590 |
|  | R putamen | 3.741 [-12.999 – 20.481] | 0.642 |
|  | R pallidum | 21.656 [-9.248 – 52.560] | 0.157 |
|  | R hippocampus | 5.353 [-29.297 – 40.0004] | 0.748 |
|  | R amygdala | 31.211 [-15.504 – 77.925] | 0.176 |
|  | R nucleus accumbens | -16.173 [-94.654 – 62.307] | 0.668 |

L, left; R, right; CI, confidence interval

Table 4. Subregional brain volume variables as predictors of BVMTR.

|  |  | Univariate analyses (controlling for age, sex and years of education) |  |
| --- | --- | --- | --- |
| Variable |  | ***β* [95% CI]** | ***P*** |
| Cross sectional volumes | L caudal anterior cingulate cortex | -0.203 [-6.934 – 6.528] | 0.950 |
|  | L caudal middle frontal gyrus | -1.836 [-5.966 – 2.295] | 0.360 |
|  | L entorhinal cortex | -7.691 [-14.928 – -0.453] | 0.039 |
|  | L fusiform gyrus | 0.688 [-2.117 – 3.492] | 0.610 |
|  | L inferior parietal lobule | 0.779 [-1.457 – 3.014] | 0.471 |
|  | L inferior temporal gyrus | 0.476 [-2.160 – 3.112] | 0.707 |
|  | L isthmus cingulate cortex | -8.334 [-23.921 – 7.253] | 0.274 |
|  | L lateral occipital cortex | -0.247 [-2.899 – 2.406] | 0.846 |
|  | L lateral orbitofrontal cortex | -3.672 [-9.264 – 1.919] | 0.183 |
|  | L lingual gyrus | -1.193 [-5.164 – 2.778] | 0.533 |
|  | L medial orbitofrontal cortex | -5.973 [-10.286 – -1.661] | 0.01 |
|  | L middle temporal gyrus | -1.285 [-3.580 – 1.010] | 0.252 |
|  | L parahippocampal gyrus | -7.807 [-14.410 – -1.204] | 0.023 |
|  | L paracentral lobule | -5.133 [-14.500 – 4.234] | 0.262 |
|  | L pars opercularis | -4.878 [-10.027 – 0.271] | 0.062 |
|  | L pars orbitalis | -2.330 [-15.138 – 10.478] | 0.705 |
|  | L pars triangularis | -4.296 [-10.267 – 1.675] | 0.147 |
|  | L pericalcarine cortex | -4.621 [-10.722 – 1.481] | 0.128 |
|  | L postcentral gyrus | -0.953 [-4.520 – 2.615] | 0.579 |
|  | L posterior cingulate cortex | 2.236 [-5.319 – 9.792] | 0.539 |
|  | L precentral gyrus | 0.263 [-2.084 – 2.610] | 0.815 |
|  | L precuneus cortex | 0.060 [-3.361 – 3.481] | 0.971 |
|  | L rostral anterior cingulate cortex | -1.017 [-6.974 – 4.941] | 0.722 |
|  | L rostral middle cingulate cortex | 0.239 [-1.534 – 2.012] | 0.779 |
|  | L superior frontal gyrus | -0.934 [-2.586 – 0.718] | 0.248 |
|  | L superior parietal lobule | 0.967 [-1.836 – 3.770] | 0.475 |
|  | L superior temporal gyrus | -1.709 [-3.585 – 0.167] | 0.071 |
|  | L supramarginal gyrus | -0.326 [-2.009 – 1.358] | 0.687 |
|  | L frontal pole | -9.765 [-23.011 – 3.482] | 0.138 |
|  | L temporal pole | -4.004 [-8.350 – 0.342] | 0.068 |
|  | L insula | -1.379 [-6.918 – 4.159] | 0.605 |
|  | R caudal anterior cingulate cortex | -1.868 [-9.843 – 6.106] | 0.626 |
|  | R caudal middle frontal gyrus | -0.328 [-5.374 – 4.718] | 0.892 |
|  | R entorhinal cortex | -0.623 [-4.579 – 3.334] | 0.743 |
|  | R fusiform gyrus | 0.703 [-1.857 – 3.263] | 0.569 |
|  | R inferior parietal lobule | -0.638 [-2.298 – 1.022] | 0.427 |
|  | R inferior temporal gyrus | -0.552 [-2.773 – 1.669] | 0.606 |
|  | R isthmus cingulate cortex | -3.291 [-15.206 – 8.624] | 0.566 |
|  | R lateral occipital cortex | 1.077 [-1.558 – 3.712] | 0.399 |
|  | R lateral orbitofrontal cortex | -2.492 [-7.389 – 2.406] | 0.297 |
|  | R lingual gyrus | 0.050 [-3.341 – 3.440] | 0.976 |
|  | R medial orbitofrontal cortex | -4.520 [-10.485 – 1.445] | 0.128 |
|  | R middle temporal gyrus | -0.616 [-2.671 – 1.439] | 0.534 |
|  | R parahippocampal gyrus | -2.772 [-15.792 – 10.249] | 0.658 |
|  | R paracentral lobule | -2.507 [-11.536 – 6.523] | 0.564 |
|  | R pars opercularis | -2.610 [-8.986 – 3.766] | 0.398 |
|  | R pars orbitalis | -2.981 [-10.830 – 4.868] | 0.433 |
|  | R pars triangularis | -3.288 [-6.719 – 0.143] | 0.059 |
|  | R pericalcarine cortex | -3.307 [-10.165 –3.551] | 0.322 |
|  | R postcentral gyrus | -1.242 [-3.789 – 1.305] | 0.317 |
|  | R posterior cingulate cortex | 0.259 [-6.625 – 7.143] | 0.937 |
|  | R precentral gyrus | -0.701 [-2.978 – 1.576] | 0.523 |
|  | R precuneus cortex | -0.297 [-4.270 – 3.677] | 0.876 |
|  | R rostral anterior cingulate cortex | -6.113 [-13.711 – 1.486] | 0.107 |
|  | R rostral middle cingulate cortex | -0.895 [-3.108 – 1.318] | 0.404 |
|  | R superior frontal gyrus | -0.501 [-2.062 – 1.059] | 0.506 |
|  | R superior parietal lobule | 1.065 [-1.351 – 3.481] | 0.364 |
|  | R superior temporal gyrus | -1.520 [-4.517 – 1.477] | 0.298 |
|  | R supramarginal gyrus | -0.343 [-3.238 – 2.552] | 0.805 |
|  | R frontal pole | -17.477 [-34.473 – -0.482] | 0.045 |
|  | R temporal pole | -4.085 [-11.852 – 3.682] | 0.281 |
|  | R insula | 1.157 [-4.338 – 6.652] | 0.661 |
|  | L lateral ventricle | -0.162 [-0.771 – 0.446] | 0.580 |
|  | L cerebellum cortex | -0.066 [-0.773 – 0.642] | 0.847 |
|  | L thalamus | 2.474 [-1.886 – 6.833] | 0.247 |
|  | L caudate nucleus | -1.796 [-8.224 – 4.631] | 0.562 |
|  | L putamen | 1.731 [-4.684 – 8.147] | 0.575 |
|  | L pallidum | -5.212 [-20.280 – 9.856] | 0.474 |
|  | Third ventricle | -1.386 [-8.819 – 6.046] | 0.698 |
|  | Fourth ventricle | 6.650 [0.226 – 13.073] | 0.043 |
|  | Brainstem | 0.293 [-2.592 – 3.179] | 0.832 |
|  | L hippocampus | 2.904 [-4.560 – 10.368] | 0.422 |
|  | L amygdala | -4.248 [-21.654 – 13.158] | 0.612 |
|  | L nucleus accumbens | 12.834 [-13.950 – 39.619] | 0.325 |
|  | R lateral ventricle | -0.292 [-0.919 – 0.335] | 0.338 |
|  | R cerebellum cortex | -0.090 [-0.779 – 0.598] | 0.785 |
|  | R thalamus | 2.763 [-0.945 – 6.471] | 0.134 |
|  | R caudate nucleus | -1.120 [-7.170 – 4.930] | 0.700 |
|  | R putamen | -0.290 [-5.814 – 5.234] | 0.913 |
|  | R pallidum | -5.565 [-17.998 – 6.867] | 0.357 |
|  | R hippocampus | 3.329 [-10.990 – 17.648] | 0.629 |
|  | R amygdala | -6.378 [-25.292 – 12.535] | 0.485 |
|  | R nucleus accumbens | -8.682 [-39.768 – 22.405] | 0.562 |
| 1-year volume changes | L caudal anterior cingulate cortex | 20.716 [-25.145 – 66.577] | 0.353 |
|  | L caudal middle frontal gyrus | 5.216 [-12.363 – 22.795] | 0.538 |
|  | L entorhinal cortex | -0.779 [-42.799 – 41.240] | 0.969 |
|  | L fusiform gyrus | 9.374 [-4.120 – 22.868] | 0.160 |
|  | L inferior parietal lobule | 4.751 [-12.230 – 21.732] | 0.561 |
|  | L inferior temporal gyrus | 6.418 [-1.744 – 14.581] | 0.115 |
|  | L isthmus cingulate cortex | -3.519 [-39.862 – 32.823] | 0.840 |
|  | L lateral occipital cortex | 1.849 [-2.884 – 6.582] | 0.420 |
|  | L lateral orbitofrontal cortex | 8.033 [-1.644 – 17.711] | 0.098 |
|  | L lingual gyrus | 6.949 [-12.244 – 26.143] | 0.454 |
|  | L medial orbitofrontal cortex | 13.734 [-7.494 – 34.962] | 0.189 |
|  | L middle temporal gyrus | 4.860 [-4.572 – 14.293] | 0.291 |
|  | L parahippocampal gyrus | 6.065 [-30.917 – 43.046] | 0.733 |
|  | L paracentral lobule | -20.675 [-46.916 – 5.566] | 0.114 |
|  | L pars opercularis | -6.240 [-25.116 – 12.636] | 0.493 |
|  | L pars orbitalis | 15.122 [-8.724 – 38.967] | 0.198 |
|  | L pars triangularis | 3.737 [-13.596 – 21.070] | 0.654 |
|  | L pericalcarine cortex | 8.564 [-20.649 – 37.777] | 0.543 |
|  | L postcentral gyrus | -1.055 [-14.457 – 12.346] | 0.870 |
|  | L posterior cingulate cortex | -12.189 [-62.987 – 38.609] | 0.618 |
|  | L precentral gyrus | 0.163 [-7.279 – 7.605] | 0.964 |
|  | L precuneus cortex | 1.107 [-13.749 – 15.963] | 0.8876 |
|  | L rostral anterior cingulate cortex | -5.942 [-40.221 – 28.337] | 0.718 |
|  | L rostral middle cingulate cortex | 4.024 [-6.864 – 14.912] | 0.445 |
|  | L superior frontal gyrus | 2.850 [-5.528 – 11.228] | 0.481 |
|  | L superior parietal lobule | 0.683 [-2.895 – 4.260] | 0.691 |
|  | L superior temporal gyrus | 1.959 [-5.056 – 8.975] | 0.562 |
|  | L supramarginal gyrus | 1.828 [-15.760 – 19.415] | 0.828 |
|  | L frontal pole | 20.582 [-49.827 – 90.991] | 0.544 |
|  | L temporal pole | -11.249 [-27.163 – 4.666] | 0.153 |
|  | L insula | 3.937 [-3.483 – 11.357] | 0.277 |
|  | R caudal anterior cingulate cortex | -2.926 [-30.878 – 25.027] | 0.827 |
|  | R caudal middle frontal gyrus | 6.232 [-11.652 – 24.117] | 0.471 |
|  | R entorhinal cortex | -4.799 [-14.041 – 4.442] | 0.287 |
|  | R fusiform gyrus | 0.729 [-17.204 – 18.662] | 0.932 |
|  | R inferior parietal lobule | 0.298 [-6.623 – 7.219] | 0.928 |
|  | R inferior temporal gyrus | 4.371 [-3.407 – 12.150] | 0.251 |
|  | R isthmus cingulate cortex | -0.808 [-27.243 – 25.627] | 0.949 |
|  | R lateral occipital cortex | 1.425 [-1.204 – 4.053] | 0.267 |
|  | R lateral orbitofrontal cortex | 18.758 [5.362 – 32.154] | 0.009 |
|  | R lingual gyrus | 9.249 [-10.502 – 29.000] | 0.336 |
|  | R medial orbitofrontal cortex | 5.352 [-10.587 – 21.291] | 0.487 |
|  | R middle temporal gyrus | 5.499 [-8.678 – 19.677] | 0.423 |
|  | R parahippocampal gyrus | 24.967 [-23.305 – 73.239] | 0.289 |
|  | R paracentral lobule | -0.092 [-16.133 – 15.950] | 0.990 |
|  | R pars opercularis | 7.689 [-27.870 – 43.248] | 0.653 |
|  | R pars orbitalis | 4.993 [-3.742 – 13.727] | 0.243 |
|  | R pars triangularis | 10.708 [-3.351 – 24.766] | 0.126 |
|  | R pericalcarine cortex | -8.068 [-27.381 – 11.246] | 0.389 |
|  | R postcentral gyrus | 0.632 [-11.462 – 12.727] | 0.913 |
|  | R posterior cingulate cortex | -0.798 [-23.872 – 22.276] | 0.942 |
|  | R precentral gyrus | -6.588 [-14.433 – 1.256] | 0.094 |
|  | R precuneus cortex | 7.577 [-3.748 – 18.902] | 0.175 |
|  | R rostral anterior cingulate cortex | 26.504 [-18.335 – 71.344] | 0.228 |
|  | R rostral middle cingulate cortex | 7.798 [-2.042 – 17.638] | 0.112 |
|  | R superior frontal gyrus | 5.036 [-1.510 – 11.582] | 0.122 |
|  | R superior parietal lobule | 3.130 [-3.429 – 9.689] | 0.327 |
|  | R superior temporal gyrus | 4.521 [-10.284 – 19.327] | 0.527 |
|  | R supramarginal gyrus | 3.373 [-13.979 – 20.725] | 0.686 |
|  | R frontal pole | -16.979 [-86.613 – 52.655] | 0.612 |
|  | R temporal pole | 5.382 [-5.895 – 16.660] | 0.327 |
|  | R insula | 1.815 [-2.056 – 5.685] | 0.335 |
|  | L lateral ventricle | 0.426 [-0.993 – 1.845] | 0.533 |
|  | L cerebellum cortex | 2.635 [-1.690 – 6.961] | 0.215 |
|  | L thalamus | 8.503 [-6.168 – 23.173] | 0.237 |
|  | L caudate nucleus | 25.517 [1.820 – 49.213] | 0.036 |
|  | L putamen | 1.137 [-19.692 – 21.966] | 0.909 |
|  | L pallidum | -3.833 [-48.709 – 41.043] | 0.859 |
|  | Third ventricle | 4.791 [-8.819 – 18.402] | 0.466 |
|  | Fourth ventricle | 1.803 [-37.671 – 41.278] | 0.924 |
|  | Brainstem | -2.235 [-10.818 – 6.348] | 0.589 |
|  | L hippocampus | 4.456 [-13.375 – 22.288] | 0.604 |
|  | L amygdala | 23.281 [-9.699 – 56.261] | 0.154 |
|  | L nucleus accumbens | 18.459 [-80.270 – 117.187] | 0.697 |
|  | R lateral ventricle | 0.445 [-1.048 – 1.939] | 0.536 |
|  | R cerebellum cortex | -0.099 [-4.263 – 4.064] | 0.960 |
|  | R thalamus | 8.801 [-7.850 – 25.453] | 0.279 |
|  | R caudate nucleus | 5.904 [-10.316 – 22.125] | 0.452 |
|  | R putamen | 3.149 [-10.090 – 16.389] | 0.621 |
|  | R pallidum | 12.279 [-12.993 – 37.552] | 0.318 |
|  | R hippocampus | 5.107 [-22.281 – 32.496] | 0.698 |
|  | R amygdala | 6.069 [-42.922 – 55.059] | 0.796 |
|  | R nucleus accumbens | 13.023 [-34.913 – 60.959] | 0.573 |

L, left; R, right; CI, confidence interval
